# Systematic evaluation of therapeutic effectiveness of Azvudine in treating COVID-19 hospitalized patients: a retrospective cohort study

**DOI:** 10.3389/fcimb.2024.1453234

**Published:** 2024-11-07

**Authors:** Yingkai Xu, Yuan Huang, Zihan Yuan, Wanbing Liu, Li Wang, Lei Liu

**Affiliations:** ^1^ Medical College, Wuhan University of Science and Technology, Wuhan, Hubei, China; ^2^ Department of Transfusion Medicine; General Hospital of Central Theater Command, Wuhan, Hubei, China; ^3^ Department of Gynaecology and Obstetrics; General Hospital of Central Theater Command, Wuhan, Hubei, China; ^4^ Department of Clinical Laboratory, The First Affiliated Hospital of Henan University, Kaifeng, Henan, China

**Keywords:** COVID-19, Azvudine, composite disease progression outcome, all-cause death, retrospective cohort study

## Abstract

**Background:**

Azvudine, a repurposed oral small molecule antiviral drug, has potential effects in combating the SARS-CoV-2 virus. However, studies on its clinical efficacy in patients with COVID-19 are still limited and controversial, and further research and validation are necessary.

**Methods:**

A retrospective cohort study was conducted on COVID-19 patients who were hospitalized in the General Hospital of Central Theater Command from 1 December 2022 to 31 January 2023. We included 132 patients treated with Azvudine and 132 controls after screening and propensity score matching. The primary outcomes including all-cause mortality and a composite outcome of disease progression such as non-invasive respiratory support, invasive respiratory support, admission to intensive care unit (ICU), and death were compared.

**Results:**

Azvudine recipients had a much lower incidence rate of composite disease progression outcome than controls (13.9075/1000 person-days versus 25.7731/1000 person-days, *P*<0.05). Azvudine recipients also possessed a lower all-cause mortality rate than controls (2.6797/1000 person-days versus 8.5910/1000 person-days, *P*<0.01). Azvudine treatment significantly reduced the risk of composite disease progression (HR: 0.37, 95% CI: 0.16-0.84, *P*=0.017) and all-cause death (HR: 0.25, 95% CI: 0.08-0.81, *P*=0.021) after adjusting potential confounding factors such as age, sex, severity of COVID-19, complications, concomitant therapy, time from symptoms to treatment, and important laboratory indicators. The subgroup analyses of composite disease progression outcome and all-cause death indicated robustness of Azvudine’s in treating COVID-19 patients in general.

**Conclusion:**

Our study demonstrates that Azvudine has a significant positive impact on the clinical recovery of hospitalized patients with COVID-19. These findings provide important support for the use of Azvudine as a therapeutic option for COVID-19, given the current divergent views on its therapeutic efficacy and its importance in public health and medical care.

## Introduction

Since its emergence in late 2019, the novel coronavirus disease (COVID-19) has rapidly developed into a global pandemic, posing a significant threat to human health and causing negative impacts on the lives and health of hundreds of millions of people worldwide (Chakraborty and Maity, 2020). This unprecedented global health crisis has prompted scientists, medical experts, and research institutions around the world to urgently explore and develop effective means of prevention and treatment, as well as drugs, to meet this challenge. Several therapeutic agents such as Nirmatrelvir/Ritonavir, Molnupiravir, and Azvudine have been authorized for the treatment of COVID-19 (Hammond et al., 2022; Jayk Bernal et al., 2022; Yu and Chang, 2022). Azvudine is the first homegrown oral anti-COVID-19 drug, and it was approved by the National Medical Products Administration on July 25, 2022. Azvudine’s approval has garnered significant attention from the medical community and provided a new weapon in the global to fight against the COVID-19 epidemic. Azvudine is a nucleoside analog that specifically acts on RNA-dependent RNA polymerase (RdRp) and that is efficiently embedded in the SARS-CoV-2 RNA synthesis process to inhibit virus replication (Zhang et al., 2021). Azvudine rapidly metabolizes *in vivo* into a 5’-triphosphate metabolite (Azvudine triphosphate) with potent antiviral activity.

In the monkey experiment, Azvudine is preliminarily demonstrated to be effective for fighting SARS-CoV-2 infection by improving lymphocyte profile, reducing viral loads, organ damage and inflammation, and protecting immune function of thymus (Zhang et al., 2021). However, the clinical therapeutic efficacy of Azvudine in fighting against COVID-19 is currently a subject of controversy in the academic community. Some studies suggest that Azvudine has significant advantages such as faster viral load reduction and higher recovery rates compared to other treatments in treating COVID-19 patients (Zhang et al., 2021; Qi et al., 2023; Ren et al., 2020; Yu and Chang, 2020). However, some studies also suggest limitations to its therapeutic efficacy are objectively existed, and further validations of Azvudine’s efficacy are urgently needed (Gao et al., 2023; Han et al., 2023; Zhu, 2023). These varying findings and differing opinions among experts highlight the necessity for additional evaluation of the clinical effectiveness of Azvudine in patients with COVID-19. The purpose of this study was to retrospectively analyze the efficacy of Azvudine in treating COVID-19 patients hospitalized in the General Hospital of Central Theater Command and to establish a solid and adequate scientific basis for clinical practice, which will better guide the therapeutic strategy of COVID-19 and improve prognosis.

## Methods

### Study design and patients

**Figure 1:**
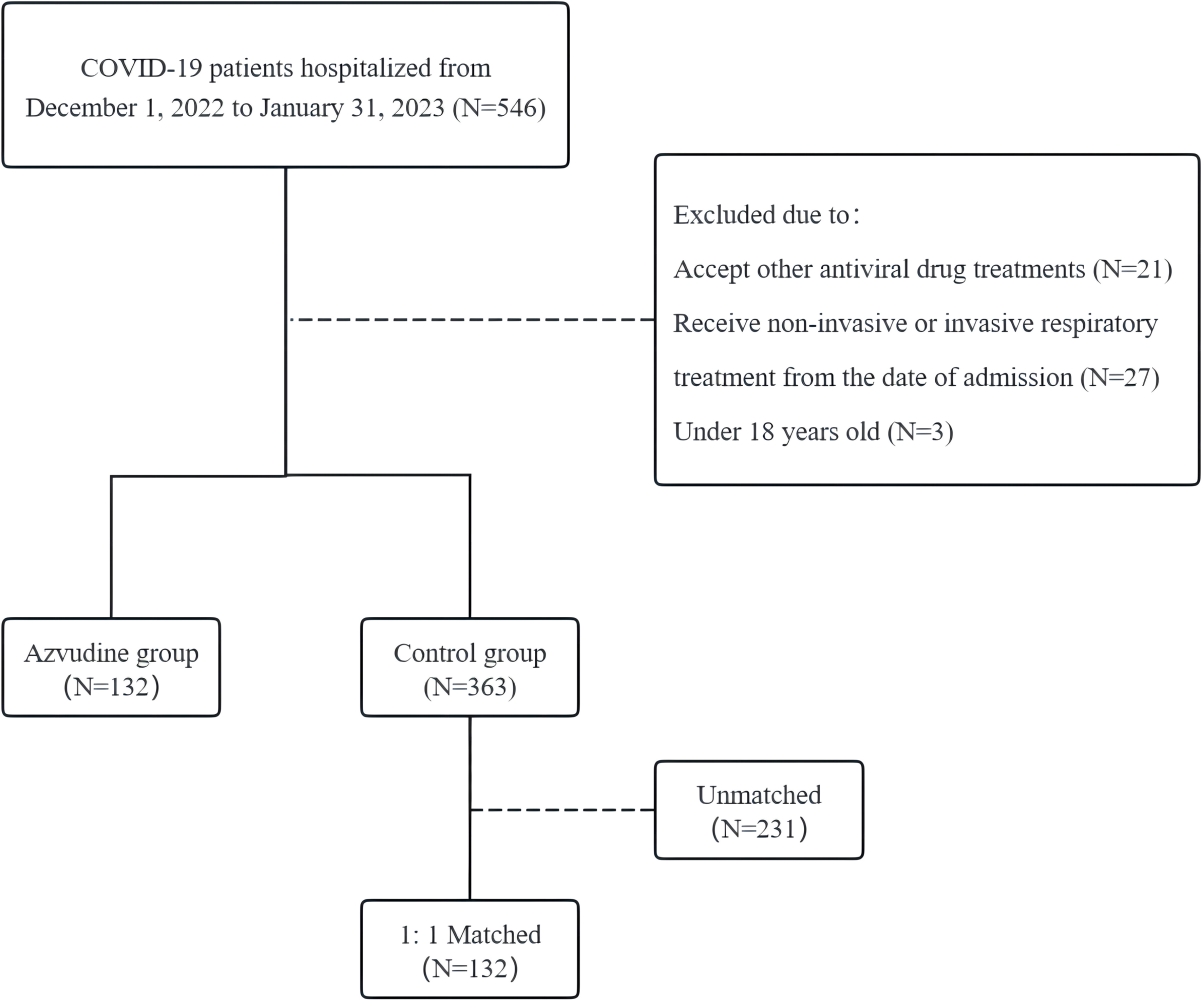
Flowchart of patient selection.

A single-center retrospective cohort study was conducted in the General Hospital of Central Theater Command from December 1, 2022 to January 31, 2023. Initially, 546 patients consecutively admitted for COVID-19 pneumonia were screened from the medical record system. Among them, 495 patients (including 132 patients with Azvudine plus standard treatment and 363 controls with standard treatment) were enrolled and met the corresponding inclusion criteria. In order to minimize the impact of confounding factors on the analysis results, we finally included 264 patients by using 132 Azvudine recipients as a benchmark and 1:1 propensity score matching (**Figure 1**). Inclusion criteria: 1) had a confirmed diagnosis of SARS-CoV-2 infection (with positive RT-PCR results or positive SARS-CoV-2 antigen detection results, or both); 2) had CT imaging findings met the standard of COVID-19 pneumonia; 3) obtained standard treatment or standard treatment plus Azvudine. The standard treatment is based on the Chinese Diagnosis and Treatment Program for COVID-19 (Trial 10th Edition). Exclusion criteria: 1) age under 18 years; 2) received antiviral medications other than Azvudine; 3) received non-invasive or invasive respiratory support on the date of admission. A patient was categorized as severe COVID-19 case on admission if any of the below clinical scenes additionally appeared: 1) respiratory rate ≥ 30/min; 2) oxygen saturation ≤ 93%; 3) arterial partial pressure of oxygen (PaO_2_)/fraction of inspired oxygen (FiO_2_) ≤ 300 mmHg (1 mmHg=0.133 kPa); 4) >50% lesions progression within 24 to 48 hours in pulmonary imaging (Chen et al., 2023). This study was approved by the medical ethics review committee of General Hospital of Central Theater Command ([2023]004-01). All enrolled patients in this retrospective cohort study were anonymous, and the requirement for informed consent was waived.

### Data collection

The comprehensive data on COVID-19 patients were collected from electronic medical information system by two investigators, including demographic characteristics, dates of admission, intervals from symptom onset to hospital admission, and initial clinical presentations. Upon admission, the disease condition of each case was evaluated based on specific criteria including respiratory rates, oxygen saturation levels, PaO2/FiO2 ratios, and the extent of pulmonary infiltrates. The past medical histories, medical orders, key laboratory test results (such as blood counts, interleukins, and calcitonin), records of ICU admissions, and dates of discharge or death were also collected. All obtained data were checked by another researcher to confirm integrity and accuracy.

### Treatment

The control group patients received the standard care outlined in the Chinese Diagnosis and Treatment Program for COVID-19 trial version 10 guidelines, mainly including 1) supportive treatments such as nutritional support, rest, maintenance of water, electrolyte balance, and acid-base balance; 2) symptomatic treatments such as oxygen therapy, fever reduction, cough and phlegm relief; 3) immunotherapy treatments such as systemic steroids and immunomodulators; 4) anti-infective treatments such as rational use of antibiotics. The patients in the Azvudine treatment group received 5 mg tablets of Azvudine orally once daily for up to 14 days on the basis of standard care. This ensured that all of these patients received the best clinical care practices at that time. The dosage and administration period were chosen based on initial evidence indicating Azvudine’s potential benefits in mitigating the effects of COVID-19. The treatment exposure period was strategically defined to commence within the first 2 days following admission to ensure the integrity of the study’s outcomes. Nasal cannula oxygen therapy was generally used to treat patients with oxygen saturation ≤ 92% or significant hypoxia symptoms. The patients who can maintain the target oxygen saturation (usually 93% -96%) in indoor air without symptoms such as difficulty breathing were often considered to stop oxygen therapy. The study carefully chose this approach to mitigate potential time bias associated with treatment initiation and admission, facilitating a more accurate assessment of Azvudine’s effectiveness. By standardizing the initiation of treatment relative to the point of hospital admission, the study aimed to reduce variability in patients’ outcomes that could arise from differences in disease progression at the time of treatment commencement.

### Outcome events

The primary outcomes were composite metrics of disease progression including non-invasive respiratory support, invasive respiratory support, admission to intensive care unit (ICU), and all-cause death. The secondary outcomes were individual disease progression related clinical manifestations such as oxygen situation. We analyzed and compared the changes in oxygen saturation between the two groups patients who did not receive oxygen therapy within 12 days after admission. We recorded the oxygen saturation of these patients every 3 days after admission. We collected data of patients from the date of admission to occurrence of any of the primary outcome events (non-invasive respiratory support, invasive respiratory support, ICU admission, or all-cause death), and discharge, whichever came first. Using this data, we were able to calculate the incidence of primary outcomes per 1,000 person-days. This allowed for a quantitative analysis of the rate of disease progression and treatment outcomes.

### Statistical analyses

This study employed a propensity score matching (PSM) approach, which incorporated several key covariates including age, sex, time from symptom onset to treatment initiation and COVID-19 severity, to achieve accurate matching. A non-release matching strategy with a caliper width of 0.2 was used. Covariate balance between groups before and after matching was assessed by calculating the Standardized Mean Difference (SMD). Covariates were considered unbalanced if the SMD value exceeded 0.1 (Austin, 2009). Furthermore, we estimated the Hazard Ratio (HR) and 95% confidence intervals (CI) for disease progression outcomes between the two groups using multifactor Cox regression models. To ensure the stability of the results across subgroups, we conducted subgroup analyses on various levels of the aforementioned covariates. Continuous variables were described as the means and standard deviations or medians and interquartile ranges (IQR) values. Categorical variables were expressed as the counts and percentages. Independent group *t* tests were applied to continuous variables that were normally distributed; otherwise, the Mann-Whitney test was used. Categorical variables were compared using the chi-square tests, while the Fisher exact test was used when the data were limited. All statistical analyses were performed using Windrush software v1.9, employing two-tailed tests with a significance level of 0.05.

## Results

### Patients’ demographics and clinical characteristics

We matched some important baseline characteristics such as age, gender, time from symptom onset to treatment exposure, illness severity, and concomitant treatments initiated on admission between two groups patients. After 1:1 propensity score matching, we finally included and identified 132 Azvudine recipients and 132 controls. **Table 1** displayed the baseline characteristics of two groups patients before and after matching. The SMD value of each of matching baseline characteristics between two groups patients was less than 0.1, indicating a better balance between them. In addition, we examined a number of comorbidities and pathophysiological indicators related to disease progression, and the results showed that no statistically significant differences were observed between two groups patients for any of these parameters. Based on these data, we concluded that these potential confounding variables did not significantly affect the study results and conclusions.

**Table 1:** Baseline characteristics before and after 1:1 propensity score matching.

Baseline characteristics	Before matching			After 1:1 propensity-score matching		
	Azvudine (n = 132)	Controls (n = 363)	SMD/P	Azvudine (n = 132)	Matched controls (n = 132)	SMD/P
Age (years), mean (SD)	71.23 (13.72)	70.95 (14.85)	SMD=0.019	71.23 (13.72)	71.45 (16.03)	SMD=0.015
Gender, n (%)			SMD=0.025			SMD<0.001
Male	87 (65.9)	235 (64.7)		87 (65.9)	87 (65.9)	
Female	45 (34.1)	128 (35.3)		45 (34.1)	45 (34.1)	
Time from symptoms onset to treatment exposure, n (%)			SMD=0.122			SMD=0.017
>5 days	94 (71.2)	238 (65.6)		94 (71.2)	93 (70.5)	
0–5 days	38 (28.8)	125 (34.4)		38 (28.8)	39 (29.5)	
Severity on admission, n (%)			SMD=0.059			SMD=0.068
Severe	35 (26.5)	87 (24.0)		35 (26.5)	39 (29.5)	
Non-severe	97 (73.5)	276 (76)		97 (73.5)	93 (70.5)	
Standard treatments, n (%)						
Supportive treatments	130 (98.4)	361 (99.4)	P =0.623	130 (98.4)	131 (99.2)	P >0.99
Symptomatic treatments	125 (94.6)	273 (75.2)	P <0.05	125 (94.6)	129 (97.7)	P =0.333
Immunotherapy treatments	122 (92.4)	268 (73.8)	P <0.05	122 (92.4)	125 (94.6)	P =0.616
Anti-infective treatments	118 (89.4)	308 (84.8)	P =0.252	118 (89.4)	115 (87.1)	P =0.702
Pulmonary complications, n (%)	49 (37.1)	137 (37.7)	P =0.888	49 (37.1)	60 (45.5)	P =0.950
Liver comorbidities, n (%)	17 (12.8)	68 (18.7)	P =0.206	17 (12.8)	25 (18.9)	P =0.136
Renal complications, n (%)	19 (14.3)	73 (20.1)	P =0.146	19 (14.3)	31 (23.4)	P =0.159
Diabetes, n (%)	37 (28.0)	118 (32.5)	P =0.458	37 (28.0)	48 (36.3)	P =0.370
Hypertension, n (%)	64 (48.5)	172 (47.3)	P =0.844	64 (48.5)	64 (48.5)	P =0.769
Lymphocyte count,10^9/L, median (IQR)	0.8 (0.5,1.2)	0.9 (0.6,1.3)	P =0.724	0.8 (0.5, 1.2)	0.9 (0.5, 1.2)	P =0.101
Procalcitonin, ng/ml, median (IQR)	0.1 (0.0,0.3)	0.1 (0.1,0.4)	P =0.803	0.1 (0.0, 0.3)	0.2 (0.1, 0.4)	P =0.359
interleukin-6, pg/ml, median (IQR)	25.2 (6.9,57.1)	25.6 (8.4,74.6)	P =0.141	25.2 (6.9, 57.1)	28.6 (8.7, 100.0)	P =0.302
D-Dimer, μg/ml, median (IQR)	0.3 (0.2,0.6)	0.3 (0.2,0.8)	P =0.365	0.3 (0.2, 0.6)	0.4 (0.2, 0.7)	P =0.536

SMD, Standard Mean Difference; SD, Standard Deviation; IQR, Interquartile Range.

### Associations between Azvudine treatment and clinical outcomes

**Figure 2:**
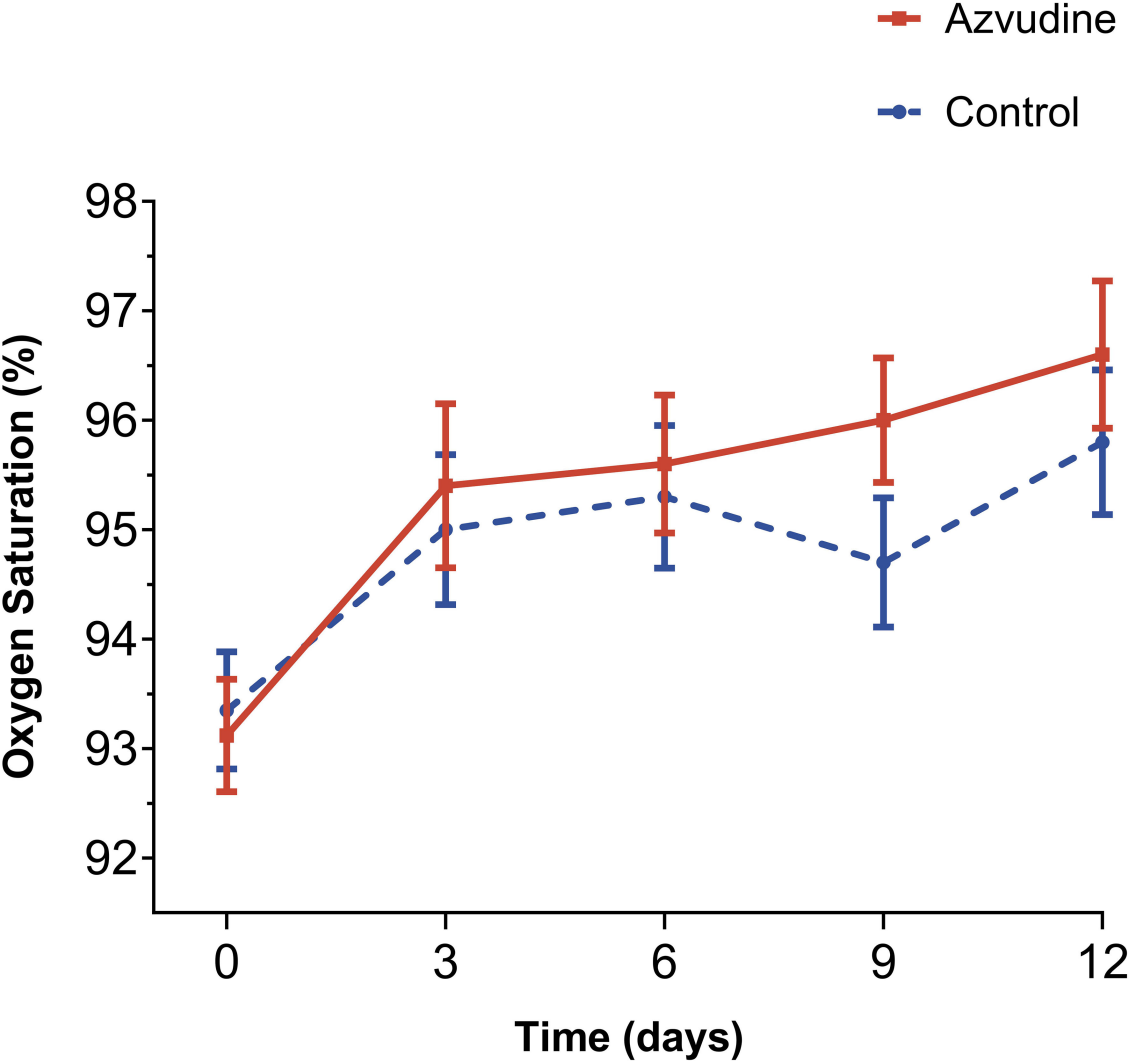
Oxygen saturation levels of Azvudine recipients and controls during hospitalization treatment. The Oxygen saturation levels of patients received standard treatment and Azvudine plus standard treatment on the 0, 3, 6, 9, and 12 days after admission for treatment were shown and compared. Day 0 (baseline) represents the first day of admission to hospital.

The proportion of patients who did not receive oxygen therapy in the Azvudine medication group and the control group was respectively 62.1% (82/132) and 58.3% (77/132), with no statistical differences (P=0.615). We compared the oxygen saturation situations of two groups patients and found that the patients treated with Azvudine overall possessed a higher oxygen saturation performance than the patients with standard treatment within 12 days after starting treatment (**Figure 2**). The oxygen saturation curve of Azvudine group patients showed a gradually increasing trend during hospitalization, while the oxygen saturation curve of controls showed a fluctuating downward and then upward trend. The oxygen saturation of Azvudine recipients was especially and significantly better within 6-12 days after treatment than controls, indicating a potential and quick improvement in respiratory function. These findings suggest that Azvudine may have a positive impact on the respiratory status and function of COVID-19 patients.

**Figure 3:**
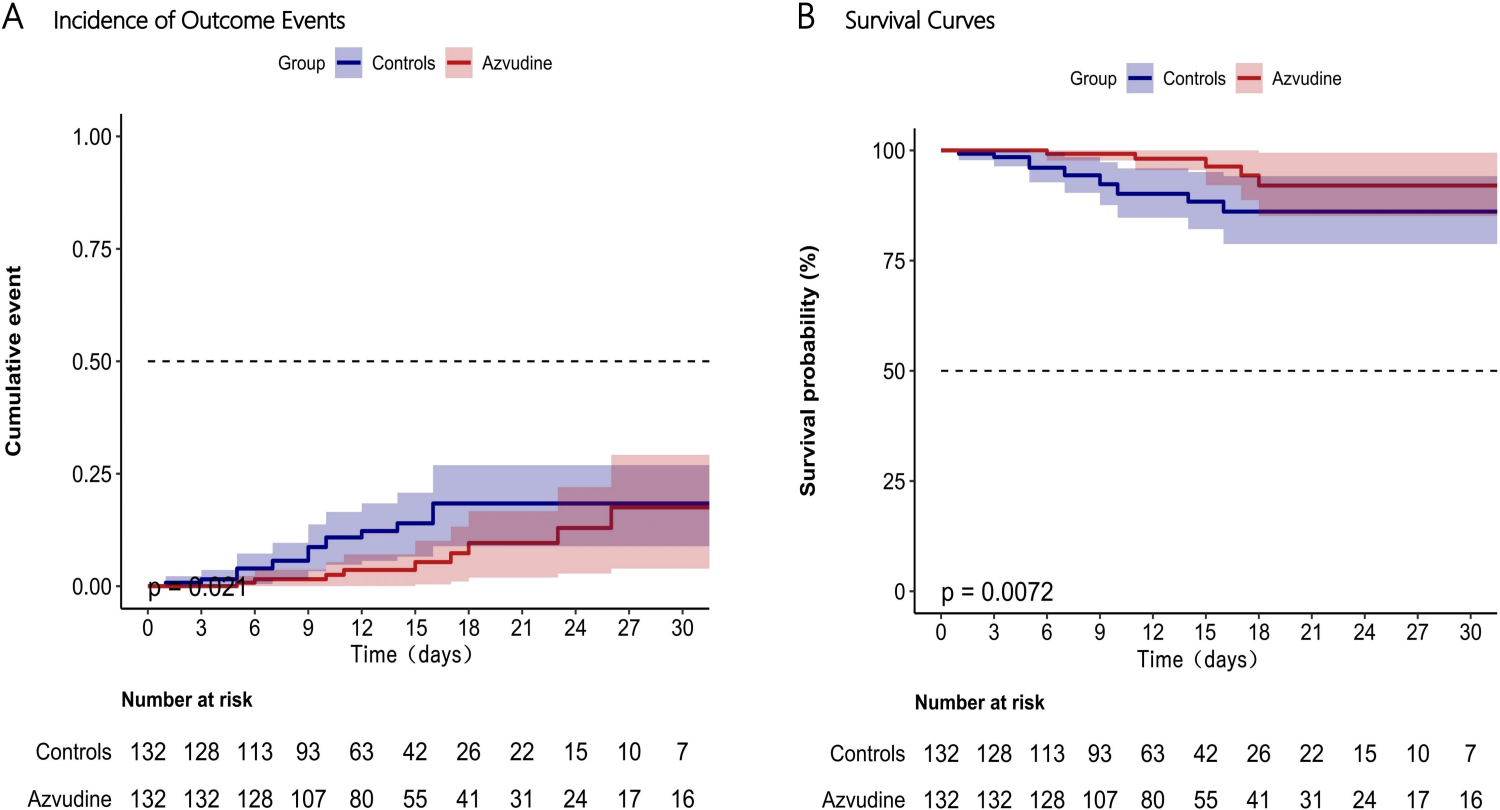
Incidence of composite disease progression outcome events **(A)** and separate all-cause death **(B)** in Azvudine recipients and controls. Day 0 (baseline) represents the first day of admission to hospital.

We found that the patients treated with Azvudine plus standard scheme had a significantly lower incidence of compound disease progression compared to the control group patients only receiving standard treatment (*P*=0.021, **Figure 3A**). The incidence rate of adverse outcome events in the Azvudine group was 13.9075/1000 person-days, while in the control group it was 25.7731/1000 person-days. Additionally, the mortality rate was significantly lower in the Azvudine group than that in the control group, with rates of 2.6797/1000 person-days and 8.5910/1000 person-days, respectively (*P*=0.0072, **Figure 3B**).

**Figure 4:**
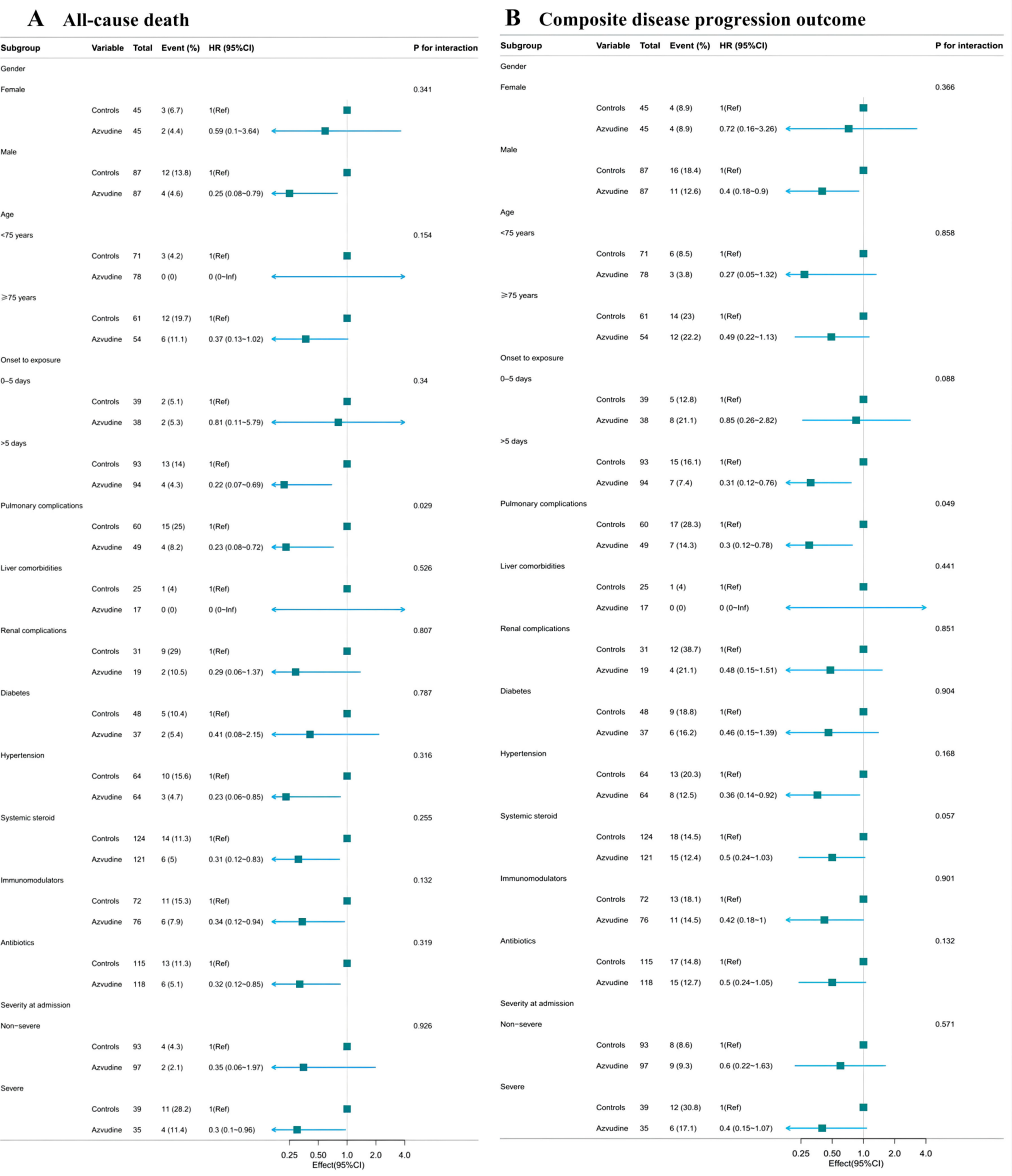
Subgroups analysis of Azvudine’s effectiveness in reducing risk of all-cause death **(A)** and composite disease progression outcome events **(B)** in patients with COVID-19.

The Cox proportional hazard model was used to further assess the association between Azvudine treatment and composite metrics of disease progression and separate all-cause death (**Table 2**). The results indicated that Azvudine treatment significantly reduced the risk of all-cause mortality (HR: 0.28, 95% CI: 0.11-0.75, *P*=0.011) and compound disease progression (HR: 0.44, 95% CI: 0.22-0.90, *P*=0.024), after adjusting for age, sex, and severity of COVID-19 (HR: 0.32, 95% CI: 0.12-0.86, *P*=0.024 and HR: 0.46, 95% CI: 0.23-0.94, *P*=0.033; respectively), or additionally adjusting for complications, concomitant therapy, and time from symptoms to treatment (HR: 0.32, 95% CI: 0.12-0.88, *P*=0.028 and HR: 0.45, 95% CI: 0.21-0.93, *P*=0.032; respectively), or further additionally adjusting for interleukin-6, procalcitonin, lymphocyte count, and D-dimer (HR: 0.25, 95% CI: 0.08-0.81, *P*=0.021 and HR: 0.37, 95% CI: 0.16-0.84, *P*=0.017; respectively). In addition, the subgroup analyses showed that use of Azvudine was significantly associated with a reduced risk of all-cause death (**Figure 4A**) and composite disease progression (**Figure 4B**) in almost all subgroups of key variables except for pulmonary complications. This result was highly consistent among the subgroups. It was also observed that Azvudine had a more pronounced therapeutic effect on patients with following characteristics such as male, severe, time from onset to treatment more than 5 days, combined hypertension, and taking accompanying treatment including systemic steroid, immunomodulators and antibiotics.

**Table 2:** Hazard analysis of Azvudine treatment on the all-cause death and composite disease progression outcomes.

Variable	Model 1^a^		Model 2^b^		Model 3^c^		Model 4^d^	
	HR (95% CI)	P	HR (95% CI)	P	HR (95% CI)	P	HR (95% CI)	P
All-cause death	0.28 (0.11~0.75)	0.011	0.32 (0.12~0.86)	0.024	0.32 (0.12~0.88)	0.028	0.25 (0.08~0.81)	0.021
Composite disease progression outcomes	0.44 (0.22~0.90)	0.024	0.46 (0.23~0.94)	0.033	0.45 (0.21~0.93)	0.032	0.37 (0.16~0.84)	0.017

a Model 1 was unadjusted.

b Model 2 was adjusted for age, sex, and severity of COVID-19.

c Model 3 was adjusted for complications (diabetes, hypertension, cardiovascular comorbidities, and other comorbidities), concomitant therapy (steroids, Immune preparations, antibiotics), and time from symptoms to treatment plus model 2.

d Model 4 was adjusted for interleukin-6, procalcitonin, lymphocyte count, and D-dimer plus model 3.

HR, Hazard Ratio; CI, Confidence Interval.

## Discussion

The research on Azvudine’s effectiveness in treating COVID-19 has yielded promising initial results, as demonstrated by some key studies in this field. A notable study by Ren et al. utilized a randomized, open-label, controlled trial design and showed that Azvudine significantly accelerates the transition to a nucleic acid-negative state in patients with mild to moderate COVID-19 and is demonstrated to be a significant advancement in therapeutic options in comparison to standard antiviral treatments (Ren et al., 2020). A randomized clinical trial corroborated these findings and showed that Azvudine can not only reduce the time to nucleic acid-negative conversion but also effectively decreases viral load and facilitates more rapid viral clearance comparing with a placebo group (da Silva et al., 2023). However, the promising results’ scope and applicability are somewhat limited by the small sample sizes used in these studies. It is important to note that the past investigations primarily focus on the timing of nucleic acid conversion, which is an important metric but does not encompass all aspects of the impact of COVID-19. These limitations raise questions about the generalizability and reliability of the observed outcomes, which underscores the need for further research involving larger cohorts to validate these preliminary findings.

Our study conducted a comprehensive examination of Azvudine’s clinical efficacy in treating hospitalized COVID-19 patients. We analyzed the impact of Azvudine on critical outcome events such as non-invasive respiratory support, invasive respiratory support, ICU admissions, and all-cause death, in addition to nucleic acid conversion times. We defined non-invasive respiratory support, invasive respiratory support, ICU admission, and death as composite disease progression outcomes. The results indicated that Azvudine significantly reduced the risk of composite disease progression outcomes and all-cause death in hospitalized patients with COVID-19. The effectiveness of Azvudine was further confirmed by adjusting several potential factors such as age, sex, disease severity, complications, concomitant therapy, time from symptoms to treatment, and some laboratory indicators. The results of subgroup analysis also showed that most of point estimates of HRs for baseline covariates and comorbidities fell to the left of 1, which demonstrates that Azvudine has stable and positive impacts on reducing mortality rate and slowing down disease progression. Thus, we may consider that Azvudine has a positive influence on the clinical recovery of hospitalized patients with COVID-19 on the basis of adopting the current standard COVID-19 treatment plan in China. Additionally, we found that Azvudine exhibited a more pronounced protective effect in male and critically ill patients with COVID-19. This observation is quite consistent with the conclusion from the retrospective cohort study by Yuming Sun et al. (Sun et al., 2023). The thymus gland, located in the human chest, is the major immune organ and is responsible for circulating T lymphocytes that play a crucial role in host immunity (Kellogg and Equils, 2021). One of the major blood abnormalities in severe COVID-19 is SARS-CoV-2-induced lymphopenia, indicating a compromised immune system (Rizk et al., 2020; Yang et al., 2020). In addition to its antiviral effects, Azvudine has immune-targeting properties and is unique among known RdRp inhibitors. It simultaneously inhibits SARS-CoV-2 replication in the thymus and promotes host T-cell immunity against the virus, a mode of action that is considered to be a dual-phase chemo-immune antiviral therapy that may be applicable to viruses that target the immune system (Zhang et al., 2021). This might explain the strong protective efficiency of Azvudine in treating severe hospitalized patients with COVID-19 and preexisting conditions. However, the potential mechanism by which male patients with COVID-19 and benefit better from Azvudine requires further investigation. Our research presented evidence indicating that Azvudine may have a significant role in the treatment of COVID-19, particularly for critically ill patients. The insights from our study can assist healthcare professionals in refining their therapeutic strategies to meet the specific needs of severely affected individuals. Additionally, our findings reveal a noteworthy variation in the effectiveness of Azvudine based on gender. This highlights the need for further investigation into how biological differences may impact the drug’s pharmacodynamics. Such research is crucial for advancing personalized medicine and ensuring that diverse patient groups receive tailored and effective treatment based on their unique physiological makeup.

The World Health Organization recommends the use of Nirmatrelvir/Ritonavir for the treatment of non-critically ill COVID-19 patients at high risk of severe illness and hospitalization, such as unvaccinated, elderly or immunosuppressed patients in its guidelines for the management of COVID-19. A number of existing studies also support the efficacy of Nirmatrelvir/Ritonavir in patients with COVID-19 (Hammond et al., 2022; Bai et al., 2024; Najjar-Debbiny et al., 2023; Reis et al., 2023; Yan et al., 2022). However, significant drug-drug interactions (DDIs) may occur when used in combination with other drugs due to the complex pharmacological mechanism of Nirmatrelvir/Ritonavir (Dresser et al., 2000). A study by Aurélie Grandvuillemin et al. showed a significant association is existed between Nirmatrelvir/Ritonavir use and the risk of drug-induced liver injury (DILI) in patients with COVID-19 (Grandvuillemin et al., 2023). Liver injury is common in COVID-19 patients (Vujčić, 2023; Yu et al., 2021). When making treatment decisions, clinicians should be aware of the potential impact on liver function, especially in patients with pre-existing liver disease. In the study by Mei-Ping Chen et al., Azvudine is the only drug to show a significant reduction in ALT and AST levels from baseline (Chen et al., 2024). Furthermore, our study also displayed that Azvudine has good therapeutic effects in the COVID-19 patients with underlying diseases.

Despite the promising outcomes, our study acknowledges several limitations that warrant caution. Firstly, this single-center cohort analysis was conducted exclusively in the General Hospital of Central Theater, and therefore the findings may not be broadly applicable. The characteristics of the specific patient demographics in this study may not be representative of the wider population encountered in various geographic locations and healthcare settings. Secondly, our research design’s retrospective nature introduces additional complexities, such as the potential for selection and information biases, despite efforts to mitigate these through techniques like propensity score matching. The absence of data on critical socio-economic, lifestyle, and patient adherence factors limits our comprehensive understanding of the myriad influences on disease progression and response to treatment. Thirdly, our study did not address the long-term efficacy and safety profile of Azvudine, including potential delayed adverse effects. This is an important area for future research endeavors. Furthermore, literature such as the study by Rui Jiang et al. has highlighted concerns about the emergence of viral resistance to Azvudine, which is a challenge faced by many antiviral therapies (Jiang et al., 2023). This issue is driven by the rapid mutation capabilities of SARS-CoV-2 and selective drug pressure, emphasizing the critical need for ongoing surveillance and investigation to maintain the effectiveness of our antiviral strategies over time.

In summary, our findings contribute valuable knowledge to the battle against COVID-19 and support the potential utility of Azvudine in treating this global health threat. Since its emergence in late 2019, the SARS-CoV-2 virus has undergone significant mutations, leading to the development of several notable strains such as Alpha, Beta, Delta, and Omicron. Each of these strains exhibits distinct characteristics regarding their transmission, potential impact on disease severity, and the effectiveness of various treatment options. Our research focused specifically on the Omicron variant. However, it is important to acknowledge that the applicability of our findings to other strains may be limited due to the unique attributes of each variant. Our study provides strong support for the use of Azvudine in treating COVID-19, despite its limitations. However, further research is needed to fully understand its effectiveness against the evolving landscape of SARS-CoV-2 mutations. Subsequent research should aim to cover a broader geographic area, include a larger number of participants, and adopt a prospective study design to evaluate Azvudine’s efficacy in diverse populations and various clinical scenarios. This approach will validate the initial promising results and contribute to refining and optimizing treatment protocols, particularly for critically ill patients. It is increasingly likely that SARS-CoV-2 will continue to circulate among humans for a considerable duration. Experts increasingly agree that COVID-19 may become endemic, meaning it will remain present in the global population. This suggests that individuals with significant risk factors may continue to be vulnerable to severe outcomes from COVID-19 infections. Therefore, it is crucial to develop more effective strategies to manage the epidemic and mitigate its public health impact. To overcome these challenges, a collaborative effort from the global scientific community is necessary. This requires emphasizing the need for collaborative research, data sharing, and innovative solutions to pave the way for more effective treatments.

## Data Availability

The data are available from the authors on reasonable request. Requests to access these datasets should be directed to LL, liulei890207@163.com.
